# Mobster: accurate detection of mobile element insertions in next generation sequencing data

**DOI:** 10.1186/s13059-014-0488-x

**Published:** 2014-10-28

**Authors:** Djie Tjwan Thung, Joep de Ligt, Lisenka EM Vissers, Marloes Steehouwer, Mark Kroon, Petra de Vries, Eline P Slagboom, Kai Ye, Joris A Veltman, Jayne Y Hehir-Kwa

**Affiliations:** Department of Human Genetics, RadboudUMC, P.O. Box 9101, 6500 HB Nijmegen, the Netherlands; Department of Molecular Epidemiology, Leiden University Medical Centre, Leiden, The Netherlands; The Genome Institute, Washington University, St Louis, Missouri USA; Hubrecht Institute, KNAW, Utrecht, The Netherlands; Department of Clinical Genetics, Maastricht University Medical Centre, Maastricht, The Netherlands

## Abstract

Mobile elements are major drivers in changing genomic architecture and can cause disease. The detection of mobile elements is hindered due to the low mappability of their highly repetitive sequences. We have developed an algorithm, called Mobster, to detect non-reference mobile element insertions in next generation sequencing data from both whole genome and whole exome studies. Mobster uses discordant read pairs and clipped reads in combination with consensus sequences of known active mobile elements. Mobster has a low false discovery rate and high recall rate for both L1 and *Alu* elements. Mobster is available at http://sourceforge.net/projects/mobster.

**Electronic supplementary material:**

The online version of this article (doi:10.1186/s13059-014-0488-x) contains supplementary material, which is available to authorized users.

## Background

Mobile elements (MEs) or transposable elements are DNA sequences that can be autonomously copied or moved through the genome, yet their highly repetitive sequence structure makes them difficult to detect. In addition to being a major evolutionary driver in changing the genomic architecture, MEs have also directly resulted in pathogenic variation in a number of human diseases by inserting into functionally important regions and disrupting gene function [1,2]. MEs can be classified into two different classes depending on their mode of transposition. Class I retrotransposons travel through an RNA intermediate by copy and paste, while class II DNA transposons have a DNA intermediate and generally move by cut and paste. Together these elements make up the majority of the human genome, with estimates in the range of 45% to 69% of the human genome sequence belonging to one of these transposons classes [3,4].

Currently only a few MEs remain active or ‘hot’ in the human genome, all of which belong to the retrotransposon class and include the autonomous L1 family (6 kb, 500,000 copies), the non-autonomous *Alu* (300 bp, 1,000,000 copies) and SVA (2 kb, 3,000 copies) families [5-8]. These ME families continue to change the genomic architecture by inserting into new regions in the DNA, transducing DNA, shuffling exons, and creating processed pseudogenes. Even ancient and inactive relics of transposition have a major contribution to genomic variation as their sequence homology can lead to unequal crossing over, resulting into deletions or duplications of DNA between two ME copies [9].

Transposition of MEs often occurs in the germline or during early embryogenesis. The first disease-causing ME insertion (MEI) in humans was found in exon 14 of the FVIII gene in two patients with hemophilia A [10]. Since then over 90 disease-producing MEIs have been found, consisting of 60 insertions of *Alu* elements, 25 insertions of L1s, and seven insertions of SVA [8]. Furthermore MEs are known to play a role in cancer development and tumor-specific MEI events have been found in several studies [11-13].

To identify polymorphic MEIs (pMEIs), both targeted and next-generation sequencing (NGS) analysis have been developed. Previous attempts to computationally detect pMEIs in human NGS data generally use discordant read pairs or clipped reads to identify pMEIs. Hormozdiari *et al.* modified VariationHunter to characterize polymorphic *Alu* insertions [14], while Ewing and Kazazian developed a pipeline for detecting polymorphic L1 insertions [15]. Tea [13] and RetroSeq [16] can use clipped reads in addition to discordant pairs to fine tune the breakpoints of the MEI event. Finally an unpublished pipeline from Stewart *et al.* can use a split-read method to detect pMEIs in longer 454 reads in addition to a paired-end approach for paired-end Illumina data [17].

We present a novel method, named Mobster, which is able to detect active non-reference MEIs with high accuracy in both WGS and WES data. Furthermore our method is not limited to a specific family of MEI events but is able to detect all families of active MEI events. Our method outperforms existing tools on a public human dataset, as well as simulation data with varying coverage. We then applied Mobster to a variety of NGS data types which include a paired-end WGS dataset, a paired-end WES dataset, and a single-end WES dataset, and performed PCR validation experiments.

## Materials and methods

### The mobster method

**Figure 1:**
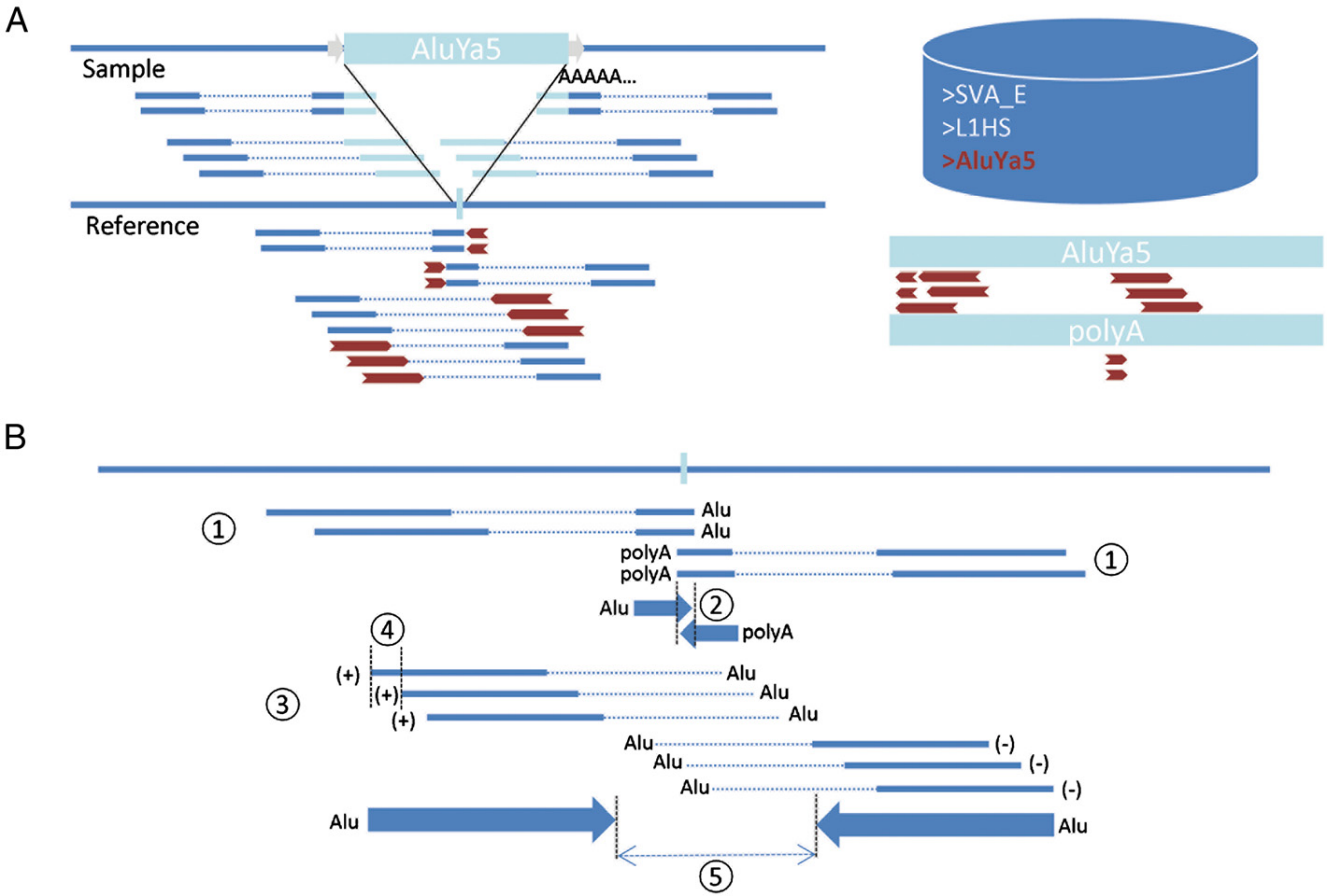
**Overview of the Mobster algorithm. (A)** In the first phase discordant ends (long red arrows) and clipped ends (short red arrows) are extracted from the BAM file when, respectively, the mate or the unclipped end is mapped uniquely to the reference. Subsequently these reads are mapped to the mobilome and investigated for having a polyA/T tail. **(B)** After mapping, all mates and unclipped sequences (anchors) belonging to unambiguously mapped *Alu*, L1, SVA, or HERV-K reads are identified. Anchors of clipped reads are clustered separately from anchors of discordant reads. (1) For clipped clusters, anchors should be: (i) supportive of the same ME family or same polyA/T stretch; (ii) clipped on the same side; and (iii) clipped within a few bp of each other. (2) The 5′ clipped cluster (arrow pointing to right), consisting of right-clipped reads, and 3′ clipped cluster (arrow pointing to left), consisting of left-clipped reads, are indicative of the same MEI event when: (i) they support the same ME family or one of the clusters supports a ME family and the other cluster supports a polyA/T tail; and (ii) they overlap by a maximum of 50 bp (allowing for TSDs) or are separated by a maximum of 20 bp (allowing for target site deletions). (3, 4) Discordant pair anchors, are clustered when: (i) they map to the same strand; (ii) are supporting the same ME family; and (iii) have start positions, which are within a specified neighborhood distance (4). (5) Forward strand anchors form 5′ discordant clusters; reverse strand anchors form 3′ discordant clusters. Discordant clusters from the 5′ and 3′ are indicative of the same MEI event when they overlap by maximal 50 bp or are within a user-defined window size. When possible, clipped clusters are merged with discordant clusters.

Mobster uses a combination of discordant read pairs and clipped reads in binary alignment (BAM) files to search for candidate active non-reference MEI events (Figure 1). Read pairs are considered to be discordant when: (1) the orientation of the mapped reads differs from the expected orientation; (2) the distance between mapped reads differs significantly from the median insert size; (3) reads are mapped to different chromosomes; or (4) one read is mapped, while the other read is unmapped. Discordant pairs which have at least one uniquely mapped read are used to anchor the possible insertion event. The mates of the anchoring reads are then mapped to a custom but configurable library of known active ME consensus sequences (mobilome, Additional file 1: Table S2). When a discordant pair contains two uniquely mapped reads, both reads are mapped to the mobilome, subsequently excluding reads which both map to a ME. If the BAM file contains clipped reads, the clipped sequences of uniquely mapped reads are also mapped against the mobilome and investigated for a polyA or polyT stretch. Anchoring reads are tagged as either unmapped or according to the mapping of their mate or clipped sequence to a ME family in the mobilome (*Alu*, L1, SVA, or HERV-K). If the data have been generated using paired-end libraries, first the discordant pair anchors supportive for the same ME family are clustered together: anchors which are within a user-specified neighborhood distance and map to the same reference strand are clustered together. Subsequently forward-strand and reverse-strand clusters indicative of the same MEI event are joined together if possible, creating both double clusters (support on 5′ end and 3′ end of putative MEI event) and single clusters. In addition to discordant pairs, clipped reads indicative for MEI are also clustered based on whether they are clipped on the right side (5′ clipped cluster) or left side (3′ clipped cluster) and map to the same ME family or to the same homopolymer (A or T). 5′ Clipped clusters and 3′ clipped clusters are joined together when both clusters map to the same ME family or when one of the clusters is indicative for a polyA or polyT stretch and the other is indicative for a specific ME family. Finally the clusters containing anchors from discordant pairs and clipped clusters are joined together. Breakpoints are estimated from the inner borders of the 5′ clipped and 3′ clipped cluster or when not available from the inner borders of discordant pair clusters. For single cluster predictions with no available clipped reads, breakpoints and prediction window borders are estimated from the insert size distribution, calculated by Picard’s CollectInsertSizeMetrics [18], and the length of the cluster itself. To avoid predicting MEIs already present in the reference, all predictions are filtered with a prediction window within 90 bp of an annotated MEI of the same ME family as the predicted MEI. In single-end libraries only clipped reads are used to predict MEI events.

Default filtering of predictions is based on events with less than five supporting reads. Supporting reads are defined, in the case of split reads, as reads that partially map uniquely to the reference genome, and partially to a ME or polyA tail. In the case of discordant reads, they refer to reads in which one end of the read pair uniquely maps to the reference genome and the second end maps to a mobile element. Furthermore data presented here are generated using Mobster with 2GB memory using 8 2.67GHz CPUs, with the exception of the CEU trio for which 8GB memory was used a comparison between the resource usage of Mobster and additional MEI identification tools is presented in (Additional file 1: Table S3).

### The mobilome and annotations

A mobilome reference database was made by selecting a subset of RepBase consensus sequences of 54 MEs thought to be still active in humans [5]. Sequences were extracted from RepBase version 17.3 [19] and include sequences from the *Alu*, L1, SVA, and HERV-K families (see Additional file 1: Table S2).

Mobster’s predictions were annotated with previously reported pMEIs found in dbRIP [16] and with novel events reported in healthy tissue and predicted *in silico* by Stewart *et al.* [17], Hormozdiari *et al.* [14], and Lee *et al.* [13] using a 50 bp window based on the prediction boundaries of Mobster’s calls. When necessary, hg18 coordinates of previously predicted pMEIs were converted to hg19 using the UCSC liftOver tool. To determine whether predictions coincide with a known gene and gene components the refSeq genes for hg19 were downloaded from UCSC. Predicted MEIs were annotated with refSeq genes using ANNOVAR [20], reporting one gene component per prediction. If a prediction overlapped with multiple gene components, for example a coding exon and an intron, priority was given to components in the following order: (1) exons, splice sites; (2) ncRNA; (3) UTRs; and (4) introns.

### Insertion and orientation bias

A UCSC custom track containing only introns was created from the refSeq genes, including alternative transcripts. Using an in-house strand-aware python script all first and last introns were extracted from the resulting BED file. With bedtools 2.15 [21] sort and merge three sets of non-redundant regions were created: all introns, first introns, and last introns. To avoid bias from genes with one intron populating only one of the subsets, these introns were included in all three sets. The hypothesized probability of a MEI inserting into the first intron of a gene was based upon the genomic size of all non-redundant first introns divided by the total size of all non-redundant introns. This hypothesized probability was tested against the observed fraction of intronic MEIs in first introns using binomial testing. Orientation of MEI events is determined by the detection of a poly A tail: 3′ detected poly A is considered the plus (+) strand, while 5′ poly A is considered minus (-) strand. The orientations of human genes were extracted from refSeq genes from UCSC.

DNA sequence insertion bias around the breakpoints was assessed for MEI events having target site duplications and consistent clipping positions of clipped clusters. For plus strand and negative strand insertions a 13 bp reference subsequence was extracted around the clipping position of the 3′ clipped cluster and the 5′ clipped cluster, respectively. Sequence logos were created for plus strand insertions and minus strand insertions separately with WebLogo 3.3 [22].

### Benchmarking with CEU trio

The performance of Mobster was benchmarked against predictions made with four different algorithms (RetroSeq [16], Tea [13], Tangram [23] and, alu-detect [24]) using a publically available data. This dataset consists of high coverage (>60X) CEU trio (NA12878 female child, NA12891 father, NA12892 mother) available through [25]. Published PCR MEI validations were used to compare the recall of mobster with other tools [17]. The MEI call sets for RetroSeq [16], Tea [13] and Tangram were downloaded from [26], while alu-detect version 1.3 was run using default settings on NA12878. Alu-detect failed to complete analysis on NA12891 and NA12892. Mobster was run requiring a minimum of five supporting reads, a neighborhood distance of 200 bp, and a maximum distance between single discordant clusters of 600 bp in order for them to be joined. Clipped sequences needed to be at least 35 bp long with a minimum average base quality of 20, while the read may not be clipped at the other end for more than 7 bp, after which only double cluster predictions were considered. *De novo* calls were defined as calls in the child not overlapping with a call in the parents within a 50 bp merging window for Mobster and a 200 bp for the other algorithms. Similarly the overlap between Mobster and the PCR validated events was based on a 50 bp window, whilst 200 bp was allowed for the other algorithms and matching ME family predicted. Different merging windows were used for a fairer comparison between the tools: on average the prediction windows made by Mobster were larger than those made by the other tools. By increasing the merging window for these tools, the recall of the PCR validated events was increased and false *de novo* calls were reduced. Furthermore the whole exome data for this CEU trio were downloaded from the same ftp site for comparison between detection of MEI events with whole genome sequencing data. The whole exome capture regions were obtained from [27].

### WGS and WES experimental datasets

Whole genome paired-end sequencing data were obtained by sequencing the DNA from healthy Dutch monozygotic twins to an average depth of 40X (EGAS00001000877), using the Illumina HiSeq platform (Illumina, Inc., San Diego, CA, USA) and paired-end libraries (Table 1) and aligned to the human reference (hg19) using BWA version 0.5.7-5 [28] with default settings. Mobster was then run, requiring a minimum of five supporting reads, a neighborhood distance of 200 bp, and a maximum distance between single discordant clusters of 600 bp in order for them to be joined. Clipped sequences needed to be at least 35 bp long with a minimum average base quality of 20, while the read may not be clipped at the other end for more than 7 bp. Putative MEI reads were mapped against the mobilome using MOSAIK v2.1.33 [29], allowing up to 10% mismatches. Similarly whole exome paired-end sequencing data were obtained for a trio and a fourth unrelated individual sequenced to high depth (95X) using a paired-end library with the Illumina HiSeq platform (EGAS00001000852) and aligned by BWA 0.5.9 [28] using default settings and the same settings for Mobster and MOSAIK as for the WGS data. Finally 101 trios of an intellectual disability patient cohort were sequenced with the SOLiD 4 system, generating 50 bp single-end reads with an average depth of 40X [30]. The short reads were mapped in colorspace to the human genome (hg19) reference using Life Technologies proprietary software, BioScope version 1.2, which uses an iterative alignment strategy. This iterative alignment strategy, clips bases at either the 5′end or 3′end of the read on each iteration, to map the read if global alignment of the read against the reference was not successful. A maximum of 15 bp hard-clipping was allowed for the 5′ end, while a maximum of 25 bp hard-clipping was allowed for the 3′ end.

**Table 1:** **Characteristics of the three different experimental datasets used to test MEI identification with Mobster**

	**Whole genome paired-end**	**Whole exome paired-end**	**Whole exome single-end**
Number samples	2	4	300
Average depth of coverage	40X	95X	40X
Read length	100 bp	90 bp	50 bp
Sequencing platform	Illumina	Illumina	SOLiD

### Validation experiments

Eleven random MEI events were selected for validation from the WGS dataset representing the following categories: (1) novel MEI calls predicted by Mobster and a second algorithm developed at the Leiden University Medical Center (LUMC) (unpublished); (2) novel MEI calls predicted by Mobster alone; (3) MEI calls predicted by previous *in silico* tools or existent in dbRIP and predicted by both Mobster and the LUMC algorithm; (4) MEI calls predicted by Mobster and existent in dbRIP or predicted by previous *in silico* tools. Similarly 10 random MEI events from the WES paired-end data were selected for validation. PCR primers were designed with a minimum of 50 bp up- and downstream the estimated insertion breakpoint.

Prior to the validation of MEI events, all primers and PCR conditions were tested and optimized using control DNA ensuring correct amplification. For MEI events with a predicted insertion size smaller than 1 kb and insertions estimated to be larger than 1 kb, different amplifications kits were used, being REDtaq readymix (Sigma-aldrich) and RangerDNA Polymerase (Bioline), respectively (PCR conditions available upon request). Subsequently, PCR amplicons were checked on agarose gels. Amplicons consistent with the presence of a hetero- or homozygous insertion were analyzed by capillary (Sanger) sequencing using routine procedures. Sequences obtained were analyzed using Vector NTI. The class of ME was determined by using a Blat search of the inserted sequences in the UCSC browser.

## Results

MEs play an active role in modifying genomic structure. However, due to their highly repetitive nature they are difficult to detect using short-read NGS technologies. We have developed a novel method (Mobster) to detect active non-reference MEI events in both whole exome and whole genome, based on single-end and paired-end sequencing data. The accuracy of Mobster was tested on both simulation (see Additional file 1: Results) and experimental human NGS data.

### Accuracy based on simulation data

The accuracy of Mobster was first tested on WGS simulation data with depths ranging from 10X to 160X. These results show a very high sensitivity (99.1%) with as little as 10X coverage for homozygous MEI events reporting no false positives (Additional file 1: Figures S1-2). At the highest coverage (160X), sensitivity is marginally improved to 99.9%, with one false positive. Paired-end WES simulation show a markedly lower sensitivity, ranging from 52.7% to 85.4% at 10X to 160X, respectively. This reflects the difficulty of identifying MEI events in small exonic capture regions, likely influenced by the simulation capturing on target but not near target sites. Overall the positive predictive value of the algorithm is good, ranging from 98.5% to 99.9% at the highest and lowest coverage, respectively.

### Accuracy based on CEU trio

The accuracy of Mobster was first tested on a WGS, high coverage, public dataset consisting of the CEU trio (NA12878, NA12891, NA12892) and compared against previous results. Tea, Mobster and RetroSeq detected around 1,200 to 1,250 *Alu* and L1 events in NA12878, while Tangram and alu-detect predicted between 1,550 and 1,620 events (Additional file 1: Table S4 and Additional files 2, 3, and 4). The trio provides a good way to benchmark the different algorithms as expected number of *de novo* MEI events lie between 0 and 2. A higher amount of *de novo events* than expected can indicate both false positives in the child and false negatives in the parents. Mobster had the lowest percentage of *de novo* MEI events (4.5%, n = 54) in comparison to RetroSeq [16] (7.7%, n = 97), Tangram (12.4%, n = 192), and Tea [13] (14.3%, n = 172) (Additional file 1: Table S4). All of the predicted events in this trio, including those which were inherited, were then compared with PCR validated events **(**Table 2). Of the 1,029 *Alu* events validated across the three individuals, Mobster detected 1,015 (98.7%), in comparison to 98.1% by Tangram, 97.8% by RetroSeq, 95.1% by alu-detect, and 91.1% by Tea. In addition Mobster detected 89 of the 99 (89.8%) validated L1 events in this trio, whereas Tangram detected 85.5%, RetroSeq 83.7%, and Tea 80.7%.

**Table 2:** **Percentage of PCR validated events recalled from CEU trio by the different algorithms**

	**Alu events**	**L1 events**
Nr PCR events	1029	99
Mobster	**98.7**	**89.8**
Tangram	98.1	85.5
RetroSeq	97.8	83.8
alu-detect	95.1^a^	NA
Tea	91.1	80.7

^a^Recall calculated based on NA12878 (408 PCR validated *Alu* events).

Values in bold depict best performing algorithm.

### Detecting MEIs in paired-end WGS experimental data

Mobster was then tested on WGS NGS 40X experimental data from monozygotic twins requiring a minimum support of five sequencing reads (see Methods). Furthermore clipped reads were required to have a length of at least 35 bp with a minimum average base quality of 20. We discarded MEI predictions which only had supporting reads at one side of the insertion and those for which the predicted insertion coordinates were within satellite DNA. In addition predicted events occurring within 50 bp of each other were merged. Using this strategy a total of 1,179 MEIs were identified, 1,068 of which were shared between the samples, resulting in a 90.6% overlap (Additional file 1: Figure S4a).

We hypothesize the remaining 111 private variants were in fact false negatives in the other sibling. We investigated this by pooling supporting anchoring reads data from both samples and reprocessing with Mobster. Each prediction required to have at least five supporting reads from one sample. The same filtering steps were used as for the non-pooled analysis, resulting in 1,181 called MEIs with no private variants, supporting our false negative hypothesis (Additional file 1: Figure S4b). By considering all predictions from one sibling to be true positives in the non-pooled analysis, then the estimated false negative rate for Mobster ranges between 3.6% for sibling C and 5.9% for sibling A.

**Figure 2:**
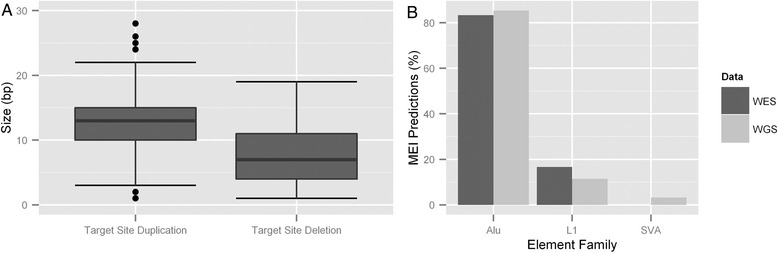
**Characteristics of detected MEI events. (A)** The predicted MEI events in the MZ twins show target site duplication sizes and target site deletion sizes characteristic of retrotransposition. **(B)** pMEI predictions in whole genome and whole exome paired-end datasets show a similar distribution pattern in mobile family origin, with *Alu* being inserted most frequently in both datasets.

The majority of the predicted MEIs show hallmarks of retrotransposition. In 954 events a reliable estimate could be made whether a MEI was associated with an indel at the site of integration. The vast majority of MEIs (n = 889) were inserted with a target site duplication (TSD), with a minority having a target site deletion (n = 57) or no indel (n = 8). The median TSD size of all MEI events was 13 bp, while the median deletion size was 7 bp (Figure 2A). Furthermore in 735 out of 753 predicted MEIs supported by clipped reads at both sides of the event a polyA tail longer than 8 bp could be detected.

The majority of MEI events detected were *Alu* in origin, followed by L1 and SVA (85.4%, 11.4%, and 3.2%, respectively), (Figure 2B). No HERV-K insertions were identified. Using a 50 bp merging window the vast majority (95.5%) of predicted events overlap with either previous *in silico* predictions in healthy individuals or with entries in the database of retrotransposon insertion polymorphisms (dbRIP). Only 436 of the 1,181 MEIs (36.9%) were predicted to occur within refSeq genes, while refSeq genes makes up 42.8% of the callable genome (Table 3). This represents a significant depletion of genic MEI events (*P* = 2.1×10^-5^, one-sided binomial testing). In addition only one MEI had a potential overlap with an exon, situated near the border of a *DSCAM* exon. Apart from the depletion of MEIs in genic regions, the locations of the MEIs seem to be randomly distributed across the genome, with 5 Mb MEI bin counts following the Poisson distribution closely (λ =2.46, considering 633 5 MB bins in a 3.1 GB genome). A strong motif signal surrounding the breakpoints of MEIs with target site duplications was observed, TTTT/A[AT], with the slash indicating the breakpoint (Additional file 1: Figure S5). This suggests the majority of events are mediated by L1 endonucleases as their most common target sequence is TTTTAA [31].

**Table 3:** **Gene components affected by MEI events in healthy individuals sequenced with WGS**

**Genomic component**	**Predictions (n)**
Genic	436 (36.9%)
Coding gene exonic	1 (0.1%)
Coding gene intronic	395 (33.4%)
Coding gene UTR5	4
Coding gene UTR3	1 (0.1%)
Non-coding gene exonic	3 (0.3%)
Non-coding gene intronic	32 (2.7%)
Non-genic	745 (63.1%)
1 kb downstream TSS	6 (0.5%)
1 kb upstream TSS	5 (0.4%)
Intergenic	734 (62.2%)

Next we investigated the orientation and insertion bias of the 427 MEIs in intronic sequences. No significant insertion bias was observed, but a slight trend was observed towards depletion of *Alu* insertions in first introns: 26.4% of 427 *Alu*s are inserted in first introns compared to an expected percentage of 29.6% (*P* = 0.18, two-sided binomial testing). While SVAs insertions (n = 12) tend to be enriched in last introns with an observed percentage of 25% and an expected percentage of 11.1% (*P* = 0.14, two-sided binomial testing) (Additional file 1: Figure S6). The orientation of 269 intronic MEIs could reliably be detected. Intronic MEIs show a significantly higher number of insertions in the opposite orientation of the gene they were inserted into; 59.5% of insertions being in opposite sense (*P* = 2.2×10^-3^).

**Figure 3:**
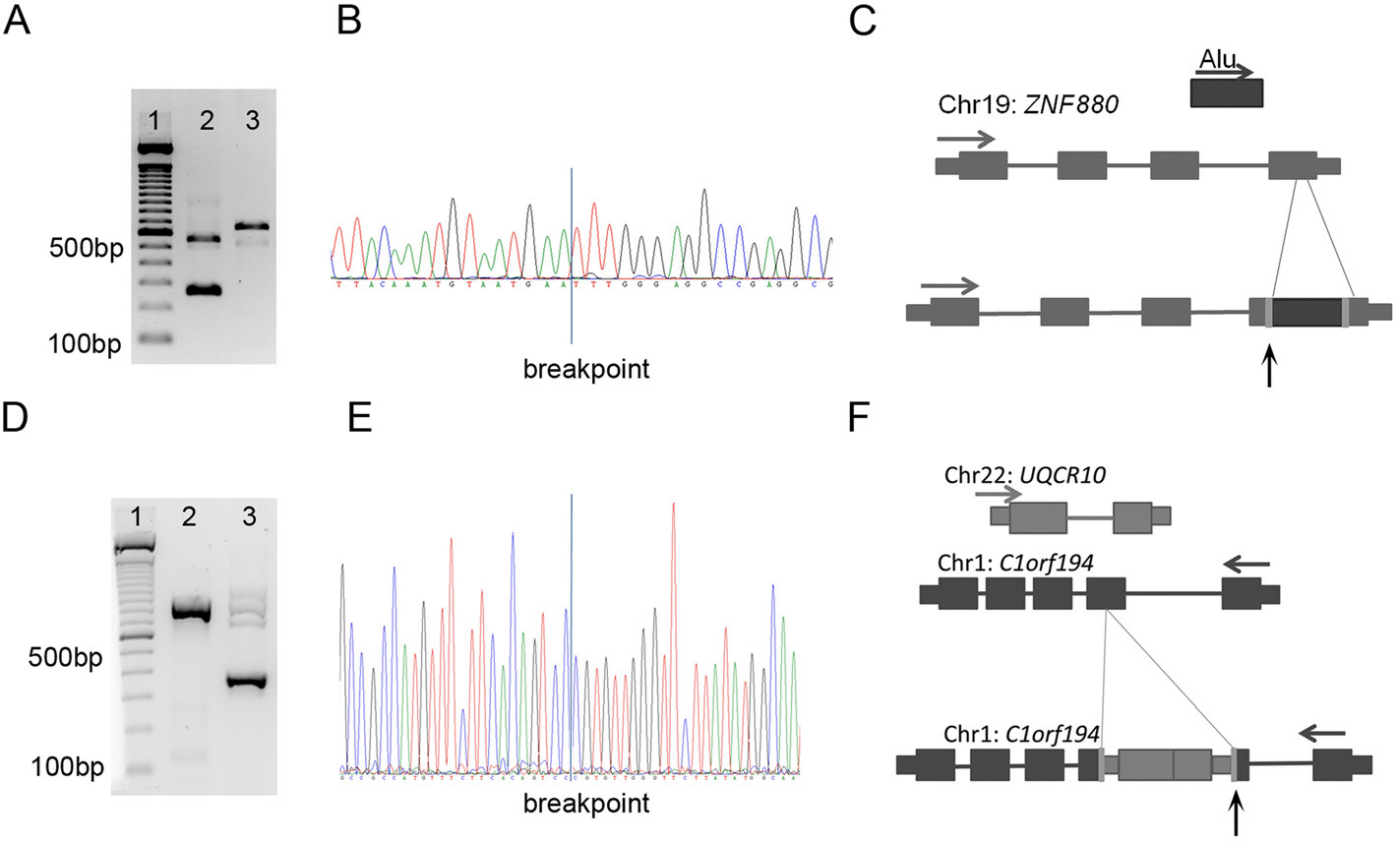
**Validation of MEI events detected. (A)** Validation of *Alu* events, bp in brackets correspond to the expected PCR product size of the wild-type allele. 1: 100 bp marker, 2: WES event10 homozygous MEI insertion (178 bp). **(B)** Sanger trace of first breakpoint. **(C)** Schema representing exonic *Alu* insertion in *ZNF880*. **(D)** Single-end exome sequencing reveals a novel processed pseudogene (*UQCR10*) insertion into the exon of *C1orf194*. 1: 100 bp marker, 2: homozygous insertion, 3: heterozygous insertion. **(E)** Sanger trace representing distal breakpoint of insertion. Distal breakpoint has been mapped to chromosome 1 between 109,650,634-109,650,635 **(F)** Schema representing the retrotransposition event.

In total 10 of the 11 events selected for validation were successfully PCR validated (FDR = 9%) (Table 4, Figure 3). Validated events were present in both heterozygous (n = 9) and homozygous (n = 1) states, and included insertion events representing the three main ME families (*Alu*, L1, and SVA). Three of the validated events were novel, previously not reported in dbRIP [16] or in *in silico* predictions of other studies [13,14,17]. In six events, Sanger sequencing of the PCR products confirmed the ME families predicted by Mobster. In the remaining five events Sanger sequences remained inconclusive.

**Table 4:** **Validation of MEI detection in WGS and WES paired-end data**

**Whole genome**								
**Chr**	**Predicted insertion point**	**MEI**	**Gene component** ^**a**^	**Gene name**	**Genotype**	**TSD**	**Novel** ^**b**^	**Validated**
chr1	60,470,596	*Alu*	Intronic	*C1orf87*	Heterozygous	Duplication		Yes
chr1	83,201,791	L1	Intergenic		Heterozygous	Duplication	Yes	Yes
chr1	93,167,519	*Alu*	Intronic	*EVI5*	Homozygous	Unknown		Yes
chr1	142,803,597	L1	Intergenic		Homozygous reference	Duplication	Yes	No
chr3	103,171,382	*Alu*	Intergenic		Heterozygous	Deletion		Yes
chr4	80,883,493	*Alu*	Intronic	*ANTXR2*	Heterozygous	Duplication	Yes	Yes
chr8	53,791,040	*Alu*	Intergenic		Heterozygous	Duplication		Yes
chr8	132,672,106	*Alu*	Intergenic		Heterozygous	Duplication	Yes	Yes
chr10	130,625,059	L1	Intergenic		Heterozygous	Duplication		Yes
chr17	43,660,608	SVA	Intergenic		Heterozygous	Unknown		Yes
chr20	29,638,569	L1	Upstream	*MLLT10P1*	Heterozygous	Duplication		Yes
**Whole exome**								
**Chr**	**Predicted insertion point**	**MEI**	**Gene component** ^**a**^	**Gene name**	**Genotype**	**TSD**	**Novel** ^**b**^	**Validated**
chr1	93,167,519	*Alu*	Intronic	*EVI5*	Homozygous	Unknown		Yes
chr2	11,426,360	*Alu*	Intronic	*ROCK2*	Homozygous	Unknown		Yes
chr3	50,879,159	*Alu*	Exonic	*DOCK3*	Homozygous	Unknown		Yes
chr5	173,036,482	L1	Exonic	*BOD1*	NA	Unknown	Yes	No
chr6	52,712,717	*Alu*	Intergenic		Homozygous	Unknown		Yes
chr9	68,415,155	*Alu*	Intergenic		NA	Unknown		Yes^c^
chr11	428,014	*Alu*	Intronic	*ANO9*	Homozygous	Unknown		Yes
chr11	112,084,617	L1	Intronic	*BCO2*	Heterozygous	Unknown		Yes
chr17	61,565,890	*Alu*	Intronic	*ACE*	Heterozygous	Unknown		Yes
chr19	52,888,055	*Alu*	Exonic	*ZNF880*	Homozygous	Duplication		Yes

^a^Overlap with gene component is determined based on Mobster’s predicted insertion window.

^b^Not overlapping dbRIP or *in silico* MEI predictions [13,14,17] within a 50 bp window.

^c^454 validation by Stewart *et al*.

On average 1,181 MEI events were detected per WGS sample of which 4.5% were novel. Ten of the 11 randomly select MEI events could be validated. MEI detection in WES produced on average 42 events per exome of which 4.8% were novel. Nine of the 10 randomly selected MEI events from the WES predictions could be validated.

TSD = target site duplication.

### Detecting MEIs in paired-end WES experimental data

To test the performance of Mobster on WES paired-end NGS data a trio and an unrelated individual sequenced to high depth using a paired-end library were analyzed (Table 1). Mobster identified on average 22 MEI events (range, 20 to 23) per individual, with a total of 87 predictions. All predictions required to have at least five supporting unduplicated reads. Support on both the 5′ end and 3′ end side of the insertion site was not required, allowing Mobster to detect insertions near the borders of the exome capture region. After merging all predictions into a unique set of MEIs, 42 loci remained. The majority of these 42 MEIs were *Alu* in origin (83.3%), with the remaining events reported to be L1 (16.7%). The predicted insertion windows of four MEIs (9.5%) overlapped with exons from *ANO5*, *ZNF880*, *DOCK3*, and *BOD1*. In addition the vast majority of events (95.2%) had previously been reported in either dbRIP or literature [13,14,17].

We focused on the parent-child trio to determine the inheritance characteristics of the MEIs. Out of the 21 insertion events in the child, 17 could be identified in at least one parent, leaving four potential *de novo* events. However these MEIs were all called near the borders of captured exonic regions, where coverage is low. Hence we hypothesized these events may have been missed in the parents by using a cutoff of five supporting reads. To test this hypothesis the sequence data from all individuals in the trio were pooled and MEI events identified, subsequent analysis of the child confirmed that these four events were false negatives in one of the parents and no *de novo* MEI events could be detected (Additional file 1: Figure S7).

A subset of 10 random events from the 42 predictions was chosen for validation. By PCR, gel electrophoresis, Sanger sequencing, or 454 sequencing nine of the 10 events were validated (Table 4, example of validated event in Figure 3A to C). These events included mostly one-sided predictions of insertions from *Alu* and L1 into intergenic, intronic, and exonic regions. For eight events Sanger sequencing of the PCR products of the insertion allele was concordant with the predicted MEI. In addition a ninth event, an *Alu* insertion into an intergenic region on chromosome 9, was previously validated using 454 sequencing [17]. The remaining 10th prediction was predicted in a parent and a child to be located in exon 3 of *BOD1*. However in both the parent and the child clipped reads were found, with ends matching perfectly to the exon/intron boundaries, suggestive for a retroposed *BOD1* copy (Additional file 1: Figure S8). Furthermore informative SNPs located on the reads supporting the MEI event in *BOD1*, suggest that the MEI event was located with the retroposed copy of *BOD1*, and incorrectly anchored into *BOD1*.

### Direct comparison of WES and WGS paired-end MEI calls in a CEU trio

Next we investigated the accuracy of detecting MEI events in WES data (Additional file 1: Table S5), by determining the overlap between MEI predictions based on WGS data from the CEU trio (NA12878, NA12891, NA12892) and WES data from the same trio. Predicted MEI coordinates from the WGS data were intersected with the capture region list, revealing only one exonic MEI event in *ZNF880*. This event, present in the WGS data of all three individuals, was also detected in the WES data of all three individuals by Mobster. The same *ZNF880* MEI event was also found and validated in our in-house experimental paired-end WES data (Table 4). Conversely all WES MEI events (n = 24) in this trio, including the predictions outside the official capture regions (n = 21), were also called in the WGS data. Demonstrating the reliability of Mobster in exome data, however more events are required in order to further investigate the influence of the capture step on detection sensitivity.

### Longer clipped reads have less chance of aligning at random to the mobilome

Due to the short read length of 50 bp for the single-end NGS data, we next investigated the required length for clipped sequences to reliably detect MEIs. Additional file 1: Table S1 summarizes how many reads out of 1,000,000 generated reads align against the mobilome using different mismatch settings in BWA. Based on these results we conclude that clipped reads of 20 bp and 1 mismatch or higher could reliably map to the mobilome.

### Detecting MEIs in single-end WES experimental data

Finally 101 parent-child trios consisting of single-end whole exome sequencing were analyzed with Mobster. Predictions required support on both sides of the insertion, with at least five supporting reads that had an average clipped length of at least 20 bases, with the clipped bases having at least an average quality of 20. In addition the clipping positions of reads on one side of the predicted MEI were not allowed to differ by more than 3 bp. Using this strategy, 89 putative exonic MEI events were found. In order to increase confidence, only predictions were considered that were present in more than one individual. Using this approach one MEI event remained, located in the second exon of *C1orf194* and predicted in five individuals.

Validation of the predicted MEI event revealed a novel pseudogene created from L1 mediated retrotransposition of *UQCR10* into *C1orf194* (Figure 3D-F), resulting in a truncated *C1orf194* protein. This retrotransposition event is estimated to occur in 15.5% of Caucasian individuals screened and present in both heterozygous and homozygous states.

## Discussion and conclusions

We present a novel method (Mobster) able to reliably detect active non-reference MEI events in both paired-end and single-end WES and WGS sequence data. The estimated FDR of Mobster based upon validation experiments is 10% or less for paired-end WGS data across *Alu* and L1 events (Table 4). These results are supported by simulation data based on different read depths (see Additional file 1: Figures S1 and S2) and benchmarking with a public NGS dataset. Previous MEI detection methods show an increase in specificity with read depths but focus on WGS data with read lengths 100 bp or longer [17]. We show that it is possible to detect MEI events in WES as well as single-end short-read (50 bp) length data, and that reads 18 base pairs long with 0 mismatches can uniquely map to the mobilome. While reads clipped to 20 base pairs have a greater than 99.99% chance of mapping uniquely when allowing for one mismatch and hence was used as the cutoff for our WES single-end analysis (Additional file 1: Table S1). This is in concordance with the predicted e-value which is calculated to be, respectively, 4.5×10^-4^ and 4.1×10^-4^.

While it is possible to detect MEI events in single-end short-read length data, both the sensitivity and specificity of Mobster improved on data with longer read lengths, and discordant paired-end information data. Many of the supporting reads for the MEI predictions were obtained by analyzing all discordant read pairs and not just those read pairs which are mapped multiple times. This demonstrates the importance of using all discordant read pairs in structural variation detection for increasing sensitivity. The 1,181 MEIs predicted in paired-end WGS data were supported on average by 47 (SD ±27) reads mapping to the mobilome or a homopolymer (A/T) stretch. The majority of these reads were originally mapped multiple times against the reference (64.6%) or clipped (24.0%). In contrast a small number of reads were originally mapped uniquely but discordant to the reference genome (8.8%), or were unmapped (2.7%). A minority of predicted MEIs were not supported by discordant read pairs on both sides of the event, but only by clipped reads (n = 47) (Additional file 1: Figure S3). By allowing these predictions, Mobsters’ sensitivity increases in genomic regions with lower coverage, hard to map regions and for heavily truncated insertion events. The majority of events detected in the single-end short read data were singletons with a higher rate of novel events, suggesting a higher false positive rate than with paired-end data. Validation of these events suggested a number of the split reads where the result of indels and not MEIs.

We determine that 4% to 5% of MEIs detected by Mobster are novel in comparison to a reference set of known MEI events consisting of dbRIP, events reported by Stewart *et al.* [17], Hormozdiari *et al.* [14], and Lee *et al.* [13]. This high overlap with previously reported MEIs suggests that Mobster has both a low false positive rate, which is supported by the FDR of the validation experiments, and that existing resources of pMEI events are currently incomplete. In comparison, low-coverage WGS in the pilot 1000 Genomes Project had a detection sensitivity of 70% to 80% for common (allele frequency >0.1) non-reference *Alu* insertion loci [17]. While high-coverage (approximately 15X to 40X) sequencing with both long (Roche 454) and short (Illumina) reads was required to achieve a per-individual sensitivity of 90% [17].

Mobster detected on average 1,100 MEI events per individual using whole genome sequencing data of which approximately 436 were genic. Previous reports suggest that approximately 5,370 non-reference MEI exist in a dataset consisting of 179 samples, of which 42% are genic, and only a small number are exonic [17]. Both our results and those previously reported indicate a depletion of MEI events in the coding regions of the genome. No significant bias towards insertion into the first or last intron of a gene was observed. However an antisense orientation bias was observed for MEIs, arguing for a selection against sense MEIs. Sense MEIs terminate gene transcription more efficiently than anti-sense MEIs [32]. Mobile element insertions into the coding exons of genes are likely to disrupt gene function and therefore face strong purifying selection. Such insertions are expected to exist only briefly in the population, as very rare insertions, which require robust high throughput detection methods for identification. Capturing these rare MEI events will allow investigation into the factors influencing ME retrotransposition rates and site preferences, prior to potentially confounding influences such as natural selection, demographic changes, and post integration rearrangements.

The majority of MEI events are consistent in structure and their integration into the genome results often in either a target site duplication or sometimes a deletion [33]. Target site duplications were observed with the majority (93%) of MEI events, while the remainder had either a target site deletion or no indel at the integration site. Deletions or duplications at the integration site occur after or during minus strand synthesis, in which a second strand nick of the target site occurs. Depending on the retrotransposon involved, the second strand nick can occur downstream, upstream, or in line with the bottom strand nick to generate target site duplications (TSDs), target site deletions, or blunt insertions [34,35]. We observed no statistically significant trend towards target site deletions or duplications dependent on the family of the MEI element (*Alu*, L1 or SVA). It has previously been observed that target site duplications and deletions tend to either be 15 nt or 9 nt in length [33], more specifically we observe that the majority of target site duplications are 14 nt in length, and target site deletions have a median of 7 nt (Figure 2B).

In addition to MEI events, the processing of novel pseudogenes is also a form of retrotransposition. We detected such gene retrotransposition events in both the single-end and paired-end WES datasets. The first and validated event involved an exonic integration site involving an L1 element, whereby *UQCR10* is inserted into exon 2 of *C1orf194* (Figure 3D to F)*.* This event was recently detected *in silico* [36]. Based on the analysis of 101 trios we estimate that this non-reference event has a minor allele frequency of 0.15 in the Caucasian population. An additional L1 retrotransposed gene was detected in the WES paired-end dataset involving *BOD1* in both the child and parental DNA. The resulting non-reference pseudogene shows evidence of exons 2 to 4 being fused (Additional file 1: Figure S8). Numerous retrotransposed versions of *BOD1* have previously been reported, the closest matching known element, *BOD1L2*, has more than 26 mismatches as well as a 6 bp gap leading to the conclusion that the event observed is novel and that BOD1 could be considered a hotspot for such events. Similar to MEI events the retrotransposition of novel pseudogenes results in structural variation which may lead to disease or result in normal genomic variation.

The development of robust algorithms to detect MEI events in NGS data is important for calculating an accurate *de novo* insertion rate for mobile elements. Previous *de novo* rates have been estimated indirectly using phylogenetic and population methods [37-39]. The relative retrotransposition rates for the three element classes *Alu*, L1, and SVA are estimated to be 0.039, 0.0056, and 0.002 insertions per genome per generation, respectively. However, phylogenetic and population methods will not detect MEIs that are lost soon after integration. The *de novo* insertion rate can be directly obtained using trio data. We present a method for the detection of MEI events in a variety of NGS data and explore some of the genomic properties of these events. The application of this method to larger cohorts would detect additional novel MEIs with potentially important functional consequences as well as retrotransposed gene events.

## Additional files

Additional file 1:
**Supplementary results, figures and tables.**
Additional file 2:
**Mobster’s double cluster calls on NA12878.**
Additional file 3:
**Mobster’s double cluster calls on NA12891.**
Additional file 4:
**Mobster’s double cluster calls on NA12892.**
