# Supplementary material for: Mobster: accurate detection of mobile element insertions in next generation sequencing data
Source: Genome Biol. 2014 Oct 28;15(10):488. doi: 10.1186/s13059-014-0488-x (PMC4228151; doi:10.1186/s13059-014-0488-x)
Supplement: Additional file 1: — Supplementary results, figures and tables. [file 13059_2014_488_MOESM1_ESM.docx]

Additional file 1

**Mobster: Accurate detection of mobile element insertions in next generation sequencing data**

Djie Tjwan Thung^1^, Joep de Ligt^1,4^, Lisenka EM Vissers^1^, Marloes Steehouwer^1^, Mark Kroon^2^, Petra de Vries^1^, P. Eline Slagboom^2^, Kai Ye^3^, Joris A Veltman^1,5^, Jayne Y Hehir-Kwa^1^

^1^Department of Human Genetics, RadboudUMC, Nijmegen, The Netherlands

^2^Department of Molecular Epidemiology, Leiden University Medical Centre, Leiden, The Netherlands

^3^The Genome Institute, Washington University, St Louis, Missouri, USA

**^4^**Hubrecht Institute, KNAW, Utrecht, The Netherlands

^5^ Department of Clinical Genetics, Maastricht University Medical Centre, Maastricht, The Netherlands

**Supplementary Results**

*Simulation data*

To assess Mobster’s accuracy across different NGS datasets, simulation data were generated to represent WGS paired-end (2x 100 bp) and WES paired-end (2x 90 bp). To simulate a WGS dataset in total 3,000 Alu-, L1-, and SVA elements were randomly and homozygously inserted *in silico* in the reference sequence of chromosome 12. Newly inserted elements needed to be at least 100 bp from reference MEs and from each other. From this artificially created chromosome, reads were simulated using *dwgsim* 0.1.10 (http://github.com/nh13/DWGSIM) with varying coverage in the range of 10x to 160x, having a constant base calling error rate of 0.02, a mutation rate of 1x10^-3^ and a random read frequency of 1x10^-4^. Simulated insert size distribution, matched those of the experimental WGS data with an median insert size of 311bp and a SD of 12bp. Simulated reads were mapped against hg19 using BWA version 0.5.9 using default settings. To simulate MEI inserted in WES paired-end data, 2,100 homozygous MEIs were inserted into exome capture regions (SureSelect Agilent V4) of chromosome 12 and at least 100 bp from each other or reference MEs and 35 bp from the border of the exome capture region. Subsequently reads were generated again using *dwgsim* 0.1.10 with median coverages in the range of 10x to 160x in the exome capture regions. Mobster was run on the simulation datasets requiring reads on both sides of the insertion (WGS paired-end) or on at least one side of the insertion (WES paired-end). All predictions required at least five supporting reads. A simulated MEI was considered detected when the prediction borders were within 90 bp of the simulated event.

**Supplementary Figures and Tables**

**Supplementary Figure 1:** Simulation experiments show that Mobster already has a high sensitivity for homozygously inserted MEIs at 10X. WES paired-end simulation experiments show lower sensitivity, mainly due to insertions near exon borders.

**Supplementary Figure 2:** Simulation experiments show a very high positive predictive value rate for both paired-end WGS and WES datasets.


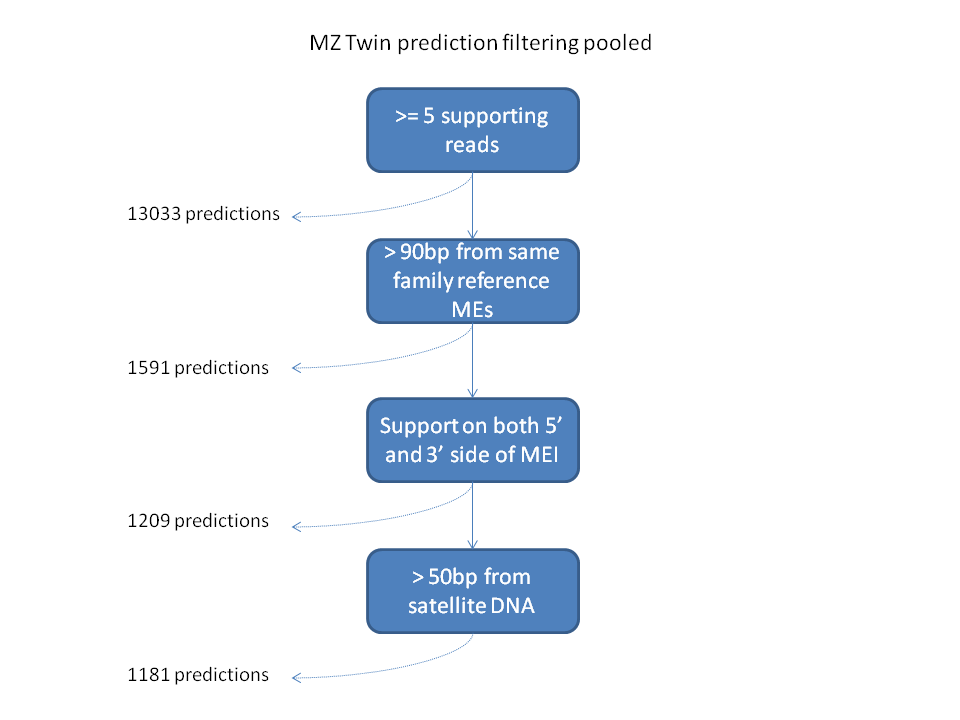


**Supplementary Figure 3:** Filtering steps used to acquire a confident MEI prediction set in the pooled analysis of the MZ twin. Most predictions are filtered because they are near a ME already annotated in the reference.

**
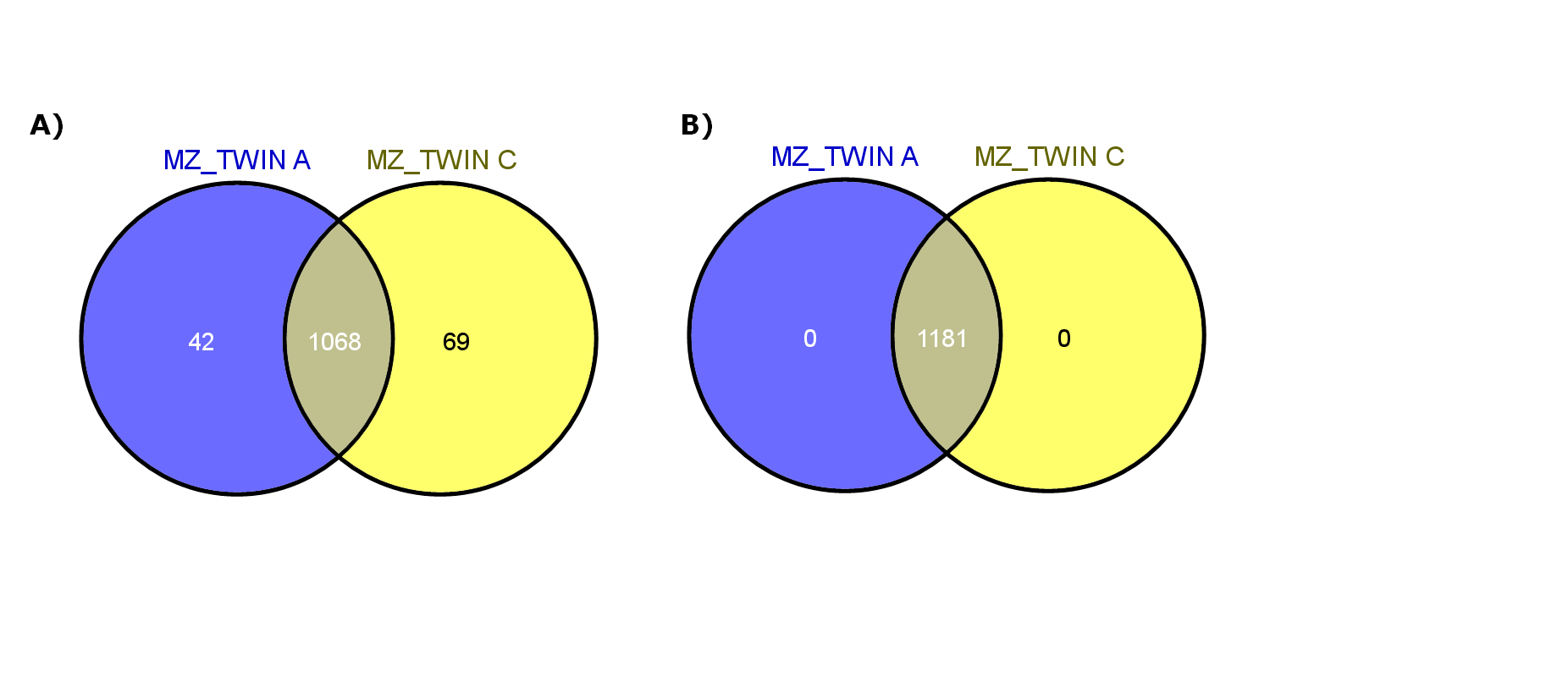
**

**Supplementary Figure 4:** Pooled analysis of the MZ twin WGS data, results in 100% overlap between the two samples and no potential *de novo* candidates. (A) Number of predictions in each sample before pooled analysis show a strong overlap of 90.6%. (B) Number of predictions in each sample after pooled analysis.


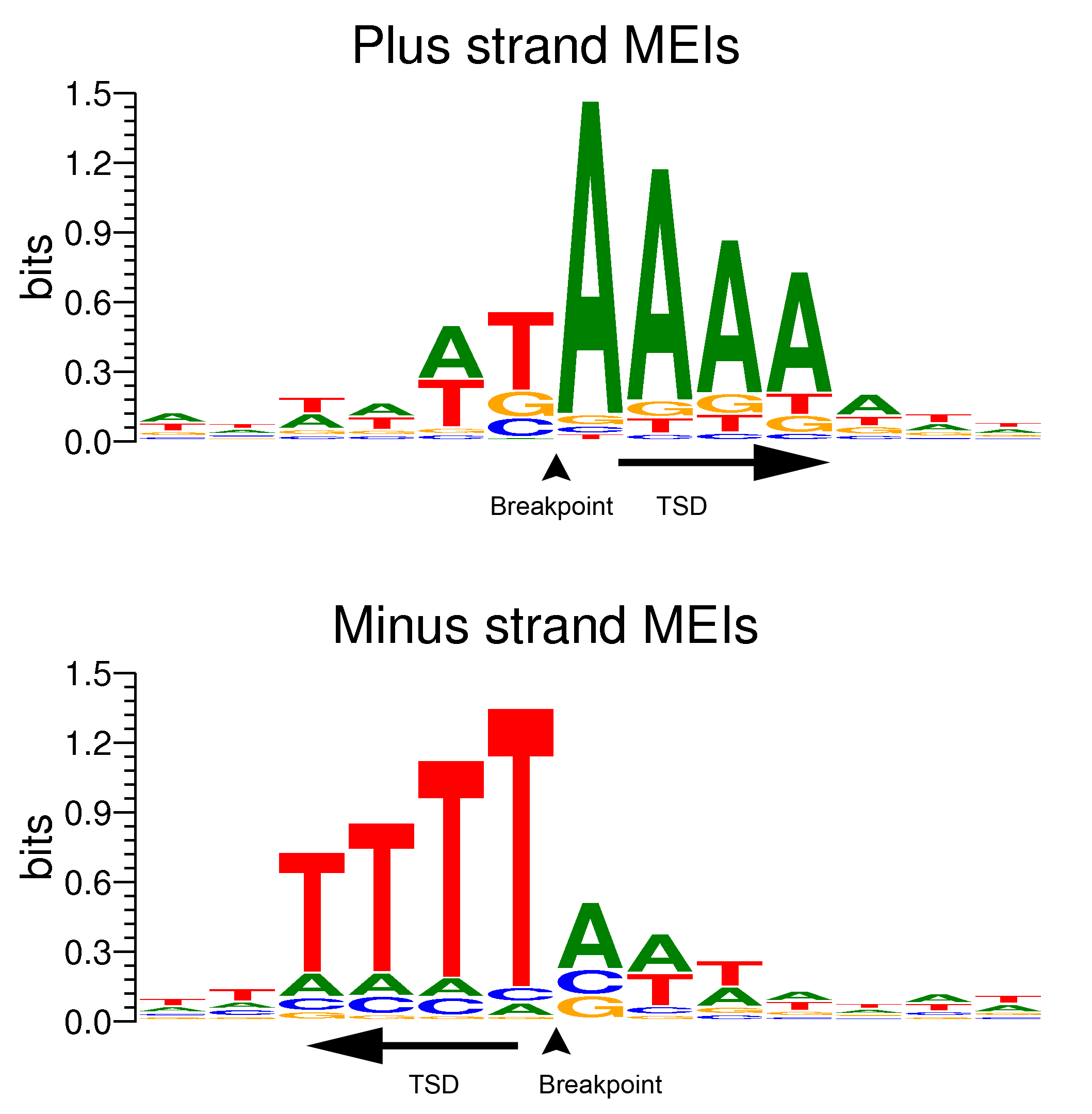


**Supplementary Figure 5:** DNA sequence motifs around breakpoints of MEIs predicted to be inserted on the plus strand and the minus strand and having target site duplications. The motifs [AT]A/AAAA and TTTT/A[AT] (slashes represent breakpoints) are indicative of L1 endonuclease mediated retrotransposition of the MEs and aid in the integration of MEs by binding to the polyA tail of the ME RNAs. ME predictions from the WGS paired-end experimental monozygotic twin data were used for this analysis and filtered for those predicted to have a target site duplication and a consistent clipping position, resulting in 206 positive strand insertions and 227 negative strand insertions.

**
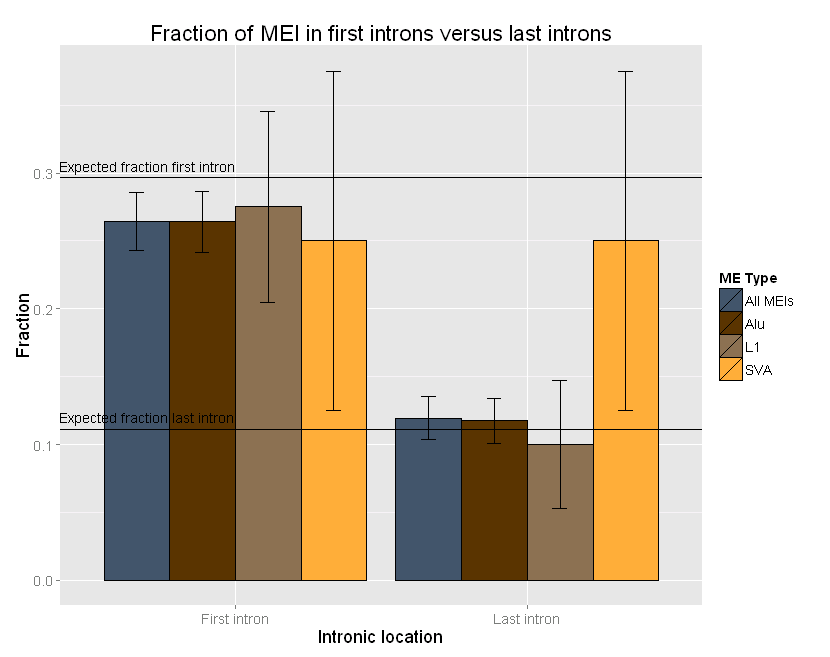
 Supplementary Figure 6:** No significant bias towards MEs to be inserted in either the first or last introns of genes was observed. However ALUs tend to be depleted from first introns, while SVAs tend to be enriched in last introns. Error bars depict the standard errors of the fractions. The expected fraction of MEs in the first intron is calculated by summing the size of all non-redundant first introns and dividing this number by the summed size of all non-redundant introns. The expected fraction of MEs in the last intron is calculated by summing the size of all non-redundant last introns and dividing this number by the summed size of all non-redundant introns.


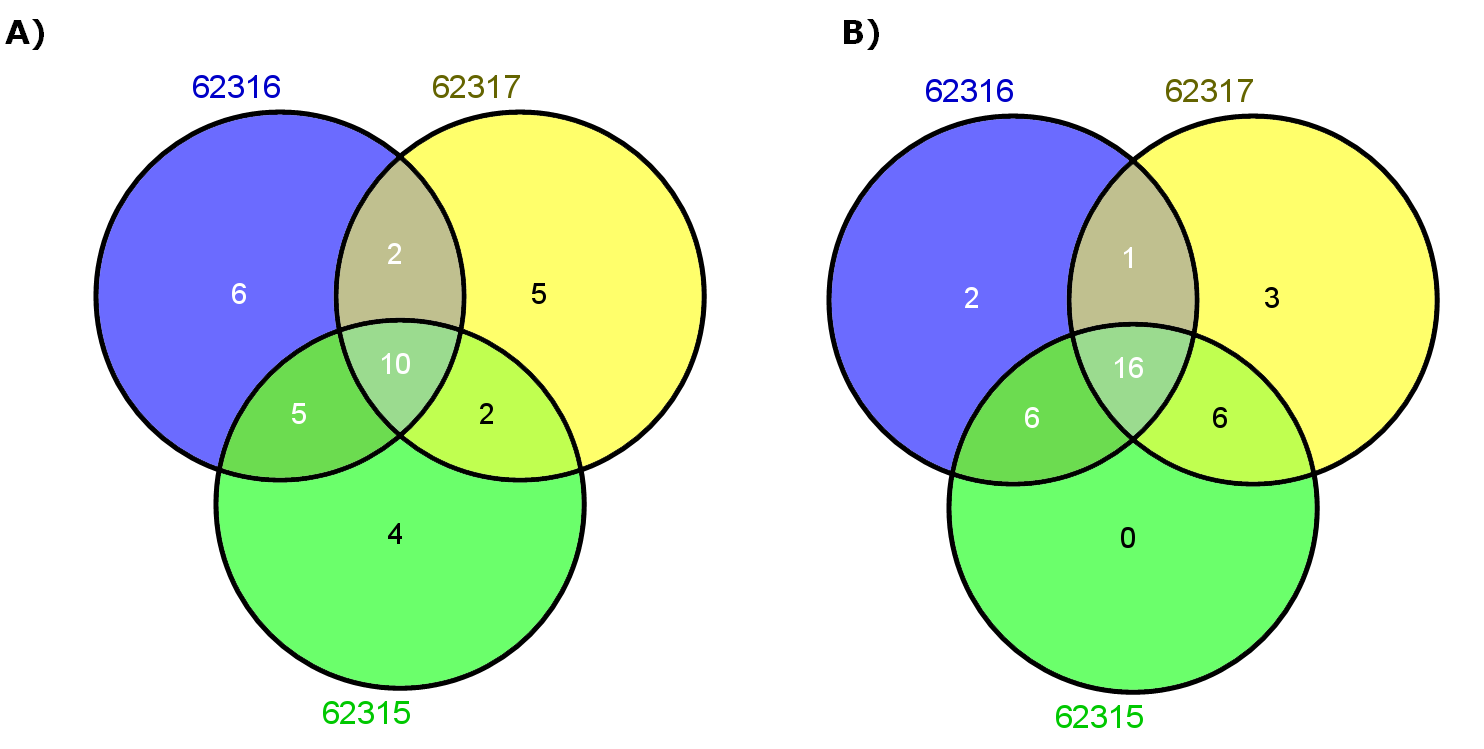


**Supplementary Figure 7:** Pooling of trio sequence data reveals no detected *de novo* MEI events in the child. (A) Detected MEI events in the trio before pooling of the sequencing data. (B) Detected MEI events in the trio after pooling of the sequencing data.


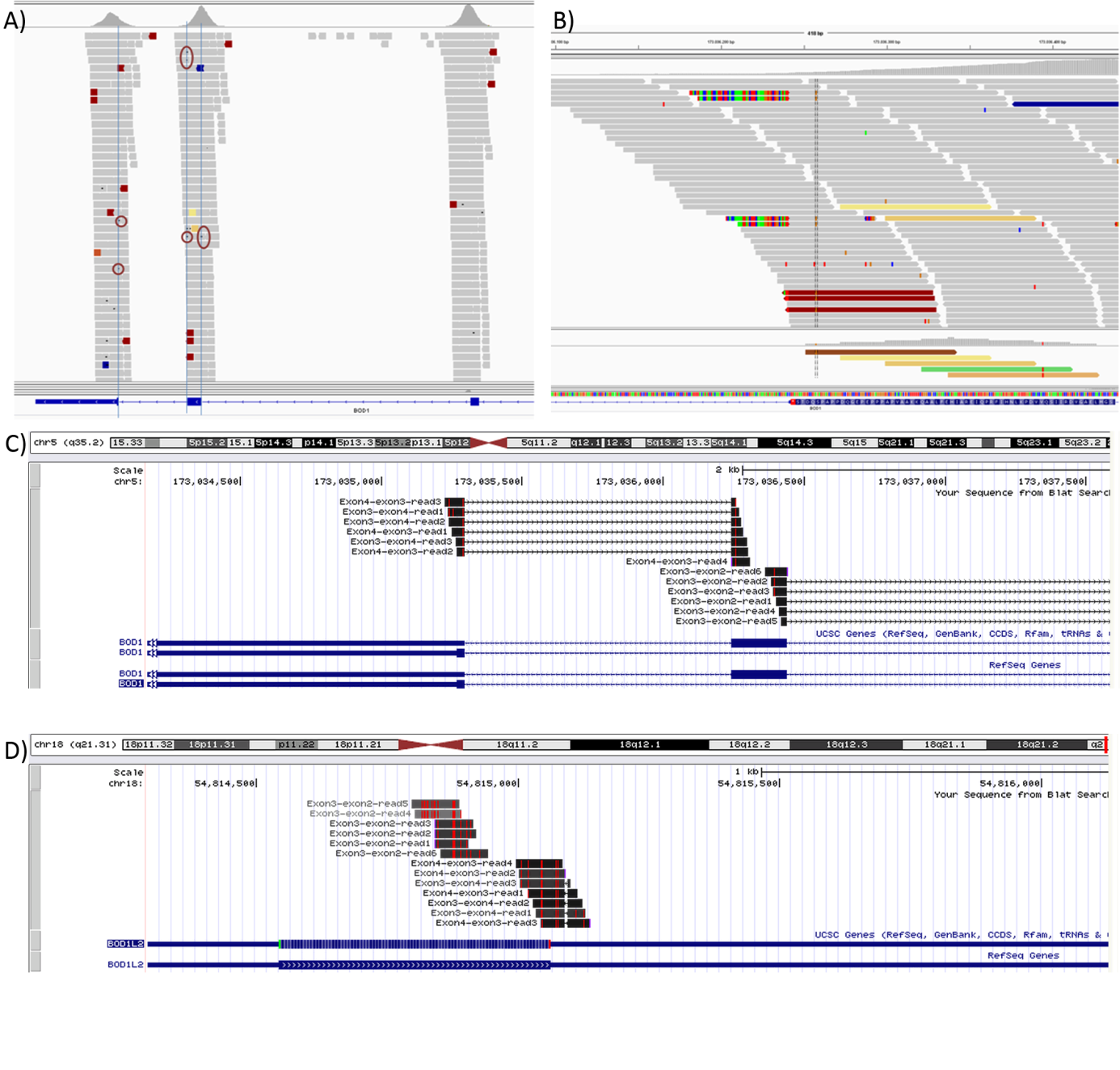


**Supplementary Figure 8:** Strong suggestion for a predicted MEI in paired-end WES to be located on a novel retrotransposed *BOD1* allele. (A) IGV view of BAM file in one of the parents. In circles the clipped positions of the reads, which all match the exon boundaries. (B) Zooming in on exon 3 of *BOD1* in IGV. In the top track we see four clipped reads (clipped sequence indicated with the sequence of red, green, blue, brown colors), with their alignment ending at the exon/intron boundary. In the bottom track anchoring reads for the predicted MEI event. One of the anchors has the same single nucleotide variant (SNV) as the clipped reads. This variant is not seen on reads overlapping both the intron and exon of *BOD1*. The other SNV seen in the anchors is also informative for the supposed retrotransposed allele. (C) When mapping the clipped reads from (B) we can see that the clipped part actually aligns to the fourth exon of *BOD1*, suggestive for a retroposed copy of *BOD1*. (D) When aligning multiple clipped reads from the BOD1 exon/intron boundaries to the closest matching retroposed copy of *BOD1* (*BOD1L2*), we observe 26 mismatches and one gap. Leading to the conclusion the predicted MEI event must be on a novel *BOD1 allele*.

**Supplementary Table 1:** Number of random reads bwa 0.5.9 maps against the mobiome using a maximum of 0 mismatches (n = 0), 1 mismatch (n = 1), or 2 mismatches (n = 2). For each read length a random set of 1,000,000 reads were generated. From read length 25 and onwards no random reads get aligned.

| Read length | n = 0 | n = 1 | n = 2 |
| --- | --- | --- | --- |
| 10 | 47,212 | 760,120 | 996,324 |
| 11 | 12,670 | 383,632 | 992,578 |
| 12 | 3,264 | 140,118 | 907,737 |
| 13 | 853 | 43,493 | 593,562 |
| 14 | 197 | 12,723 | 265,286 |
| 15 | 59 | 3,691 | 94,185 |
| 16 | 21 | 1,022 | 30,533 |
| 17 | 3 | 265 | 9,053 |
| 18 | 0 | 82 | 2,666 |
| 19 | 0 | 13 | 776 |
| 20 | 0 | 6 | 258 |
| 21 | 0 | 5 | 63 |
| 22 | 0 | 0 | 23 |
| 23 | 0 | 0 | 4 |
| 24 | 0 | 0 | 1 |
| 25 | 0 | 0 | 0 |

**Supplementary Table 2:** The mobiome consists of 54 consensus sequences extracted from RepBase 17.3 and include elements from the Alu, L1, SVA, and HERV-K families.

| **Mobile element family** | **Mobile element subfamily** |
| --- | --- |
| Alu | AluSc |
| Alu | AluSg |
| Alu | AluSp |
| Alu | AluSq |
| Alu | AluSx |
| Alu | AluSz |
| Alu | AluY |
| Alu | AluYa1 |
| Alu | AluYa4 |
| Alu | AluYa5 |
| Alu | AluYa8 |
| Alu | AluYb3a1 |
| Alu | AluYb3a2 |
| Alu | AluYb8 |
| Alu | AluYb9 |
| Alu | AluYbc3a |
| Alu | AluYc1 |
| Alu | AluYc2 |
| Alu | AluYd2 |
| Alu | AluYd3 |
| Alu | AluYd3a1 |
| Alu | AluYd8 |
| Alu | AluYe2 |
| Alu | AluYe5 |
| Alu | AluYf1 |
| Alu | AluYf2 |
| Alu | AluYg6 |
| Alu | AluYh9 |
| Alu | AluYi6 |
| HERV-K | HERV-K14CI |
| HERV-K | HERV-K14I |
| L1 | L1 |
| L1 | L1HS |
| L1 | L1PA10 |
| L1 | L1PA11 |
| L1 | L1PA12 |
| L1 | L1PA12_5 |
| L1 | L1PA13 |
| L1 | L1PA13_5 |
| L1 | L1PA14 |
| L1 | L1PA14_5 |
| L1 | L1PA15 |
| L1 | L1PA16 |
| L1 | L1PA16_5 |
| L1 | L1PA17_5 |
| L1 | L1PA2 |
| L1 | L1PA3 |
| L1 | L1PA4 |
| L1 | L1PA5 |
| L1 | L1PA6 |
| L1 | L1PA7 |
| L1 | L1PA7_5 |
| L1 | L1PA8 |
| SVA | SVA |

**Supplementary Table 3:** Computation resources used for predicting MEI events in NA12878 WGS data (number of reads is 2,873,647,625) and NA12878 WGS downsampled data (number of reads is 431,047,503). Tea, requiring hg18 BAM files, could not be run on the specific BAM file. Tangram did not finish successfully.

| **Tool** | **CPU time**  **(hh:mm:ss)** | **Wall time**  **(hh:mm:ss)** | **Memory usage (kb)** | **Virtual memory (kb)** |
| --- | --- | --- | --- | --- |
| Mobster | 8:39:24 | 6:40:04 | 8,305,780 | 23,026,612 |
| RetroSeq^a^ | 31:52:48 | 25:16:06 | 2,030,676 | 3,757,596 |
| alu-detect | 984:16:35 | 227:58:15 | 48,586,128 | 62,622,860 |
| **Downsampled BAM file (approximately 15% of total size)** | | | | |
| Mobster | 1:18:28 | 1:00:11 | 5,585,240 | 23,026,612 |
| RetroSeq^a^ | 4:02:16 | 2:57:52 | 634,392 | 1,203,428 |
| alu-detect | 130:10:12 | 21:59:48 | 11,045,556 | 12,247,904 |

^a^RetroSeq was run without the -align parameter for faster run times. Wall time with the -align parameter is 5:41:54 for the downsampled BAM file.

**Supplementary Table 4:** Number of predictions in NA12878 per algorithm and the fraction of these predictions found to be *de novo*. Lowest *de novo* rate is marked in dark gray.

| *Alu events* | | | |  |
| --- | --- | --- | --- | --- |
|  | **Predictions (n)** | **Fraction called *de novo*** |  |  |
| Mobster | 1,058 | 0.0321 |  |  |
| RetroSeq | 1,078 | 0.0510 |  |  |
| Tea | 1,037 | 0.1311 |  |  |
| Tangram | 1,326 | 0.1229 |  |  |
| *L1 events* |  |  |  |  |
|  | **Predictions (n)** | **Fraction called *de novo*** |  |  |
| Mobster | 147 | 0.1361 |  |  |
| RetroSeq | 174 | 0.2414 |  |  |
| Tea | 168 | 0.2143 |  |  |
| Tangram | 227 | 0.1278 |  |  |
| *Alu and L1 events combined* | | | | |
|  | **Predictions (n)** | **Fraction called *de novo*** |  |  |
| Mobster | 1,205 | 0.0448 |  |  |
| RetroSeq | 1,252 | 0.0775 |  |  |
| Tea | 1,205 | 0.1427 |  |  |
| Tangram | 1,553 | 0.1236 |  |  |

**Supplementary Table 5:** MEI events identified in WES data from CEU trio (NA12878, NA12891, NA12892).

| **Sample** | **Chromosome** | **Mobile element** | **Insertion point** | **Start prediction window** | **End prediction window** | **Nr supporting reads** |
| --- | --- | --- | --- | --- | --- | --- |
| NA12878 | chr4 | ALU | 78,873,809 | 78,873,789 | 78,873,829 | 5 |
| NA12878 | chr4 | ALU | 186,382,146 | 186,382,126 | 186,382,166 | 19 |
| NA12878 | chr5 | ALU | 61,857,116 | 61,857,096 | 61,857,240 | 6 |
| NA12878 | chr11 | ALU | 428,014 | 427,994 | 428,034 | 25 |
| NA12878 | chr13 | ALU | 21,894,764 | 21,894,744 | 21,894,784 | 7 |
| NA12878 | chr14 | ALU | 57,508,006 | 57,507,986 | 57,508,026 | 6 |
| NA12878 | chr17 | ALU | 61,565,890 | 61,565,870 | 61,565,910 | 14 |
| NA12878 | chr19 | ALU | 52,888,074 | 52,888,054 | 52,888,094 | 10 |
| NA12878 | chr22 | L1 | 44,324,589 | 44,324,569 | 44,324,609 | 7 |
| NA12892 | chr4 | ALU | 78,873,809 | 78,873,789 | 78,873,829 | 9 |
| NA12892 | chr5 | ALU | 61,857,118 | 61,857,098 | 61,857,138 | 13 |
| NA12892 | chr11 | ALU | 428,014 | 427,994 | 428,034 | 23 |
| NA12892 | chr13 | ALU | 21,894,764 | 21,894,744 | 21,894,784 | 9 |
| NA12892 | chr14 | ALU | 57,508,006 | 57,507,986 | 57,508,026 | 10 |
| NA12892 | chr17 | ALU | 61,565,890 | 61,565,870 | 61,565,910 | 11 |
| NA12892 | chr19 | ALU | 52,888,074 | 52,888,054 | 52,888,094 | 17 |
| NA12891 | chr4 | ALU | 186,382,146 | 186,382,126 | 186,382,166 | 16 |
| NA12891 | chr5 | ALU | 61,857,118 | 61,857,098 | 61,857,138 | 5 |
| NA12891 | chr8 | L1 | 62,115,160 | 62,115,140 | 62,115,180 | 6 |
| NA12891 | chr11 | ALU | 428,014 | 427,994 | 428,034 | 23 |
| NA12891 | chr13 | ALU | 21,894,764 | 21,894,744 | 21,894,784 | 8 |
| NA12891 | chr17 | ALU | 61,565,890 | 61,565,870 | 61,565,910 | 15 |
| NA12891 | chr19 | ALU | 52,888,074 | 52,888,054 | 52,888,094 | 16 |
| NA12891 | chr22 | ALU | 24,270,462 | 24,270,428 | 24,270,465 | 6 |
